# Supplementary material for: SyNDI: synchronous network data integration framework
Source: BMC Bioinformatics. 2018 Nov 6;19:403. doi: 10.1186/s12859-018-2426-5 (PMC6219086; doi:10.1186/s12859-018-2426-5)
Supplement: Supplementary file 5 — Files of the example presented in Additional file 4. This additional file is a zip package that contains all files used in Additional file 4. Detailed descriptions of each of these files are presented in a README file included in this zip package. (GZ 30411 kb) [file 12859_2018_2426_MOESM5_ESM.gz › SyNDITools/Meme2Fimo/meme_4.9.0_4.tar.gz!/meme_4.9.0/doc/mhmmscan.html]

mhmmscan


# mhmmscan -- not currently supported

**Usage:** `mhmmscan [options] <HMM file>
<FASTA file>`

**Description:**

`mhmmscan` searches a sequence database using a
Meta-MEME motif-based hidden Markov model (HMM) of the kind
produced by `mhmm`. This program
is similar to `mhmms`, except that
`mhmmscan`

- searches arbitrarily long sequences, and
- allows the model to match a given sequence multiple times, and
  each match is reported separately.

Each sequence-vs-model match is assigned an *E*-value, and
matches that score below a user-specified *E*-value threshold
are printed in order of increasing *E*-value.

`mhmmscan` has two modes of computing match
scores:

- *p-*value score mode.
- log-odds score mode

In ***p-*value score mode**, the search sequence can be
thought of as consisting of three steps:

1. find motif matches ("hits") with each sequence with
   *p-*values less than the user-specified **p-*value
   threshold*,
2. coalesce hits found in each sequence into "matches", where hits
   separated by more than *maxgap* positions are always
   separated into distinct matches, and
3. report matches with *E*-values less than
   *threshold*.

The three parameters **p-*value threshold*,
*maxgap* and *threshold* are described in more detail
under "Options:", below.

In **log-odds score mode**, the search can be thought of as
consisting of two steps:

1. find local matches between the model and each sequence that
   maximize the log-odds score and exceed *minscore*, and
2. report matches with *E*-values less than
   *threshold*.

The *threshold* parameter is described in more detail under
"Options:", below.

In order for *E*-values to be computed by
`mhmmscan`, at least 100 matches must be found. If there
are too few sequences in the database, or if certain other options
are made to stringent (see Options, below), too few matches may
exist for *E*-values to be computed. In this case, the results
are sorted by match score, the *E*-value column is set to
"NaN" and all matches are printed.

**Input:**

- <*HMM file*> - An HMM in Meta-MEME
  format must be provided. If the filename is given as '-', then
  `mhmms` will attempt to read the HMM from standard
  input.
- <*FASTA file*> - `mhmms` requires a
  sequence database in FASTA format.
  If the filename is given as '-', then `mhmms` will
  attempt to read the database from standard input.

**Output:**

The `mhmmscan/MCAST` output has up to three sections
containing your search results:

- Database Search Results
- Alignments
- Motif Diagrams

All three sections are always present in `MCAST` output.
The second two sections will not be present in
`mhmmscan` output unless the -fancy option was
specified.

The results in all three sections are sorted by increasing
*E*-value if possible, or by decreasing match score if
*E*-values could not be computed.

**DATABASE SEARCH RESULTS**

The "Database Search Results" section consists of lines of the
following form:

`<ID> <E-value>
<Score> <Hits> <Span> <Start> <End>
<Length> <Description>`

These fields contain, for each match found,

- **ID** - The sequence identifier, as given in the database
  file.
- ***E*-value** - The *E*-value, which is the total
  number of matches that you would expect with match scores as good
  as this match if the database contained only sequences unrelated to
  the query. Thus, a small *E*-value indicates a good
  match.
- **Score** - The match score, which is the sum of the scores
  for hits, minus the penalties for gaps.
- **Hits** - The number of hits in the match.
- **Span** - The length of the match, measured from the start
  of the first hit to the end of the last hit.
- **Start** - The position in the sequence where the match
  begins.
- **End** - The position in the sequence where the match
  ends.
- **Length** - The length of the sequence that contains the
  match.
- **Description** - The sequence descriptor. This description
  is taken from the FASTA database file, and is truncated so that the
  output fits easily on one line.

**ALIGNMENTS**

Each alignment lists the sequence identifier, match
*E*-value and log-odds score along the left. On the right, it
shows the alignment of the match with the sequence in groups of
four segments. An example segment from an alignment is given below,
followed by a description of what each line of the segment means.
(The example shows *p*-value score mode. The row of
*p*-values would be replaced by log-odds scores in log-odds
score mode. If '--motif-scoring' is not on, the row of
*p*-values or scores is absent.)

|  |  |
| --- | --- |
| hb\_P1\_element  1.5e-07  55.02 | ```                              2.4e-04                           2.4e-04            1.3e-04                              *_____+3__*                       *____-2__*         *___+1_*                              TTTTTTATGCG.......................TTTTATGACT.........CTAATCCG..................................                               TTTTTAT+ +                       TTTTAT A T         +TAATC+G           220 CGGAACATTAAAATGATTTTTATTTCTATGCTAAATCTGTTGTATTTACTTTTATAAATTTAATGTGTTTAATCTGTTCACATTTTTAAATACTTCGTATGCTATCNNNN     329 ``` |

- The bottom-most line in each segment contains 50 letters from
  the target database sequence, flanked by that segment's start and
  end locations within the entire sequence. Thus, the first segment
  would be flanked by "1" and "49", the second by "50" and "99",
  etc.
- Aligned above these 50-letter segments are the motifs
  corresponding to the hits in the match. The motifs are labeled with
  numbers in the order they appear in the query. A plus or minus sign
  preceding a hit indicates that the hit occurs on the given (+) or
  reverse complement (-) of the DNA sequence in the database. Each
  position within a motif region is indicated by a letter, and each
  gap position is indicated with a period.
- In between the sequence segment and the corresponding match
  positions is a line that indicates the degree of match between the
  motifs and the sequence. If the letter in the motif with the
  largest log-odds score appears in the sequence, then the match row
  contains that letter. If the sequence letter does not have the
  largest log-odds score, but does have a positive log-odds score,
  then the match row contains a plus sign. Otherwise, the match row
  is empty.
- The top-most row of each segment shows the *p*-value (or
  log-odds score) of each hit aligned above the start of the hit,
  depending on the score mode.

  
  


**MOTIF DIAGRAMS**

The motif diagrams section shows the matches in schematic
format. For each match, in the right two columns, it shows the
sequence identifier and the match *E*-value. On the left, it
shows the positions and spacings of the hits making up the match.
Hits are labeled with numbers corresponding to the order the motifs
were given in the query. A plus or minus sign preceding a hit
indicates that the hit occurs on the given (+) or reverse
complement (-) of the DNA sequence in the database.

**LOG-ODDS SCORES**

The log-odds scores for each motif column are created using
prior information on the letters appearing in alignment columns.
The prior information is the target frequencies [Karlin,S. and
Altschul,S.F., PNAS USA , 87, 2264-2268] implicit in a scoring
matrix. Meta-MEME can read a user-specified scoring matrix (in the
same format as used by the BLAST family of programs) from a file or
generate a PAM matrix. By default, PAM 250 is used for proteins,
and PAM 1 is used for DNA. For DNA, the "PAM 1" frequency matrix
is

```
              .990 .002 .006 .002
              .002 .990 .002 .006
              .060 .002 .990 .020
              .020 .060 .002 .990
```

Meta-MEME calculates the target frequencies *q_ij_ =
p_i_p_j_ exp(L s_ij_)* from the
scoring matrix *s_ij_* and the background letter
frequencies *p_i_* by finding the value of *L*
that makes the *q_ij_* sum to one. These target
frequencies are then used to create pseudo-frequencies to be added
to the emission frequencies of the column, following the approach
of [Henikoff,S. and Henikoff,J.G., JMB, 243, 574-578]. The
pseudo-frequency for the *i*^th^ letter is computed
as: *g_i_ = sum _j in alphabet_ (f_j_
q_ij_/p_j_)*.

The pseudo-frequencies, *g_i_*, are then combined
with the emission frequencies, *f_i_* to give
frequency estimates

*Q_i_ = (alpha f_i_ + beta
g_i_) / (alpha + beta).*
  

Finally, the log-odds score for a letter in the motif column
is computed by dividing by the background frequency of the letter and
taking the logarithm,

 *S_i_ = log(Q_i_ / p_i_).* 

In general, alpha should be proportional to the amount of
independent information in the emission frequencies. We have set it
to the constant 20. The parameter beta is arbitrary and controls
the relative importance of prior information. We set it to the
constant 10.

Our method is essentially that used in PSI-BLAST [Altschul,S.F
et al., NAR, 25:17, 3389-3402] without

1. sequence weighting, and
2. scaling for amount of independent information (alpha).

To do 1) and 2) correctly would require having and using alignment
information rather than emission frequencies as the starting
point.

**Options:**

- --gff <*file*> - Produce an output file in GFF. The output
  columns are as follows:
  - `seqname`: as given in the input seqence file.
  - `source`: `Meta-MEME`.
  - `feature`: for motif regions, `motif%d`,
    where %d is the motif ID, as assigned by MEME; for spacer regions,
    `spacer`.
  - `start`, `end`: the start and end
    positions of this region, indexed (as per the GFF spec) from
    1.
  - `score`: the *E*-value associated with this
    match
  - `strand`, `frame`: `.` (a
    period, indicating "undefined")
- --p-thresh <*p-*value threshold> - The '--p-thresh'
  switch activates *p-*value score mode with the given
  threshold. In *p-*value score mode, motif match scores are
  converted to their *p-*values. They are then converted to bit
  scores as follows:

  *S =
  -log_2_(p/T)*

  where *S* is the bit score of the hit, *p* is the
  *p*-value of the log-odds score, and *T* is the
  *p*-value threshold. In this way, only hits more significant
  than the *p*-value threshold get positive scores. The
  *p*-value threshold, *T*, must be in the range
  *0<T<=1*. This mode of scoring automatically activates
  the '-motif-scoring' feature (described below under "Advanced
  Options:") so that partial motif hits are disallowed.

  **Note**:

  - If **p-*value threshold* is too small, there may be
    few (or no) "hits", and, consequently, few (or no) matches. This
    may cause `mhmmscan` to be unable to compute match
    *E*-values, or to report no matches. Small values of the
    **p-*value threshold* may also cause the reported
    *E*-values to be inaccurate. In this case, the *E*-values
    will always be too large (conservative). The proper value for the
    **p-*value threshold* can only be determined by
    experimentation since it depends on the number of motifs, the
    information content of the motifs and the value of
    *maxgap*.
  - If **p-*value threshold* is too large, the expected
    length of a match may be longer than most of the sequences in the
    database you are searching. This will prevent `mhmmscan`
    from being able to compute *E*-values. Very low values of
    **p-*value threshold*, when search genomic DNA, tend to
    give high scores to low-complexity sequence and repeated
    elements.
- --max-gap <*maxgap*> - The '--max-gap' switch allows
  you to specify the longest gap allowed before two local matches
  will be split. Matches separated by a gap longer than
  *maxgap* will be split into two separate matches. This
  switch automatically activates the '--zselo' feature (described
  below under "Advanced Options:") so that gap emission scores are
  set to zero. It also sets the '--min-score' threshold to a small
  number (1e-6), and sets '--gap-open' and '--gap-extend' (described
  below under "Advanced Options:") to T/L, where 'T' is the
  '--min-score' threshold, and 'L' is *maxgap*.

  **Note**:

  - This switch causes `mhmmscan` to ignore the
    transition probabilities in the HMM.
  - Large values of *maxgap* combined with large values of
    **p-*value threshold* may prevent `mhmmscan`
    from computing *E*-values due to the problem described above
    in the second note for the --p-thresh switch.
- --e-thresh <*threshold*> - `mhmmscan`
  prints the matches with *E*-values below the given threshold.
  The default threshold is 10. If *E*-values cannot be computed,
  all matches are printed.
- --fancy - The '--fancy' switch turns on a more detailed output
  format that shows, in addition to the score for each sequence, the
  complete model-to-sequence match. For more details, see the
  documentation for `mhmms`.
- --width <*int*> - Specify the width (in characters)
  of each line in the output. The description of each sequence, which
  is taken from the input FASTA file, will be truncated as necessary.
  By default, the output width is 132 characters.
- --text - Print output as ASCII text (default is HTML).
- --nosort - Do not sort the output.
- --bg-file <*file*> - This switch allows you to
  specify a different background letter distribution. The background
  letter distribution is used in converting the emission
  probabilities in the model to log-odds. By default, the background
  letter distribution of the appropriate (DNA or protein) NCBI
  non-redundant database is used. With the '--bg-file' switch, the
  background distribution is read from a file whose format is the
  same as used by MEME and MAST. The file must contain one line for
  each letter in the (unambiguous) character set. Each line must
  contain the letter followed by the letter's frequency
  (probability). All other lines in the file are ignored, including
  comment lines starting with '#'. For example, *file* might
  contain:

  ```
  # tuple   frequency_non_coding
        a       0.324
        c       0.176
        g       0.176
        t       0.324
  ```

  (You can produce a file in the proper format from any FASTA
  sequence file (DNA or protein) using the fasta-get-markov utility,
  which is included with the Meta-MEME distribution in directory /bin.
  Type

  ```
                fasta-get-markov < f.fasta > f.bg
  ```

  to make a file named "f.bg" containing the letter distribution from
  FASTA file "f.fasta". )
- --allow-weak-motifs - In *p-*value score mode, weak motifs
  are defined as ones where the best possible hit has a
  *p-*value less than the *p-*value threshold. Such motifs
  cannot contribute to a match in *p-*value score mode. By
  default, `mhmmscan` rejects any search containing weak
  motifs, unless the -allow-weak-motifs switch is given. In that
  case, the search will proceed, but the weak motifs will never
  appear in any matches. **Note**:This switch only applies to
  *p-*value score mode.
- --blocksize <*value*> - Specify the number of
  letters that are read from each sequence at once. By default,
  `mhmmscan` reads in blocks of *10^7^/N*
  letters, where *N* is the number of states in the model. You
  can see the actual value being used by setting
  `--verbosity` to 2 or higher. If your system has a lot
  of memory, then you can specify a larger number of letters to be
  read at once, and vice versa.
- --synth - The '--synth' switch causes `mhmmscan` to
  generate random synthetic sequences if fewer than 10000 matches are
  found in the sequence database. These sequences will be searched
  and their match scores used to estimate the random score
  distribution. The sequences are generated using the background
  distribution specified using the '--bg' switch, above. If the
  background file contains a higher-order Markov model, the
  higher-order lines are **not** ignored when generating synthetic
  sequences.
- --progress <*value*> - Print to standard error a
  progress message after every *value* sequences.
- --verbosity 1|2|3|4|5 - Set the verbosity level of the output
  to stderr. The default level is 2.
- --noheader - Do not put a header on the output file.
- --noparams - Do not list the parameters at the end of the
  output.
- --notime - Do not print the running time and host name at the
  end of the output.
- --quiet - Combine the previous three flags and set verbosity to 1.
- --text - Do not produce HTML output. By default, mhmmscan looks
  for the Perl program "mhmm2html" and pipes its output through it to
  produce HTML. This switch turns off that conversion.

**Advanced Options:**

*The following five options are automatically set when you
specify the '-max-gap' option. If you do not use '-max-gap', you
can set these options individually.*

- --zselo - Specifying the '--zselo' switch causes the spacer
  emission log-odds scores to be set to zero. This prevents regions
  of unusual base/residue composition matching spacers well when the
  spacer emission probabilities are different than the background
  probabilities. It is particularly useful with DNA models.
- --gap-open <*cost*> - The '--gap-open' switch causes
  **all** transitions into a spacer state to be assigned a
  log-odds score equal to *cost*. Together with the
  '-gap-extend' switch, this allows you to specify an affine gap
  penalty function, overriding the gap penalty implicit in the model
  (transition probabilities into and out of gap states).
- --gap-extend <*cost*> - The '--gap-extend' switch
  causes **all** spacer self-loop log-odds scores to be set to
  *cost*. In addition, it causes all other transitions out of
  a spacer to be set to zero. Together with the '--gap-open' switch,
  this allows you to specify an affine gap penalty function,
  overriding the gap penalty implicit in the model (self-loop
  transition probabilities of gap states).
- --min-score <*minscore*> - This switch allows you to
  specify the threshold for the repeated match algorithm used by
  `mhmmscan`. Matches must have a score of at least
  *minscore* to be detected. Matches containing internal
  regions with scores less than minus 'threshold' will be split and
  reported as two separate matches.
- --egcost <*egcost*> - Scale the expected cost of a
  random gap to be *egcost* times the expected score of a
  random hit. By default, gap costs are essentially zero. The larger
  you set *egcost*, the more gaps will be penalized. This can
  only be used in conjunction with '--max-gap'. This may not be used
  in conjunction with '--min-score'.

*The following option is automatically invoked when you
specify '-p-thresh'. You can also set it when you do not want*
p-value score but want to prevent partial matches to motifs.

- --motif-scoring - Specifying the '--motif-scoring' switch
  forces all matches to motifs to be complete. This prevents matches
  to motifs from overhanging the sequence ends. It also prevents
  matches from beginning (ending) anywhere but at the start (end) of
  a motif. By default, matches can begin or end anywhere within a
  motif.

*The following options can be used in both* p-value and
log-odds score modes to control how the emission probabilities in
the HMM are converted into log-odds scores.

- --pseudo-weight <*beta*> - By default, the
  pseudocount probabilities are weighted by *beta = 10*, and
  emission probabilities in the model by *alpha = 20*. (See the
  formula above for converting letter frequencies to letter scores.)
  The weight on the pseudocount probabilities can be adjusted to any
  value greater than or equal to zero using the '-pseudo-weight'
  switch. The smaller the weight, the less effect the pseudocount
  probabilities have, and the closer the adjusted probabilities will
  be to the emission probabilities in the model.
- --pam <*distance*> - By default, target
  probabilities are derived from the *distance-250* PAM matrix
  for proteins, and from a *distance-1*
  transition/transversion matrix for DNA. With the '--pam' switch,
  you can specify a different **integer** distance from 1 to 500.
  (This can be overridden with the '--score-file' switch, below). The
  *distance-1* transition/transversion joint probability
  matrix for DNA is given below:

  ```
             A    C    G    T   
        A  .990 .002 .006 .002
        C  .002 .990 .002 .006
        G  .006 .002 .990 .002
        T  .002 .006 .002 .990
  ```
- --score-file <*score file*> - The '--score-file'
  switch causes a score file (in BLAST format) to be read and used
  instead of the built-in PAM (for proteins) or
  transition/transversion (for DNA) score file. The target
  probabilities for letters are then derived from the score file.
  Several score files are provided (including BLOSUM62) in directory
  mhmm/data. Other, user-provided score files may be specified as
  well, as long as they are in the same format.

**Bugs:** None known.

Author: William Stafford Noble
and Timothy Bailey.
